# Incidence of breast and colorectal cancer among immigrants in Ontario, Canada: a retrospective cohort study from 2004-2014

**DOI:** 10.1186/s12885-018-4444-0

**Published:** 2018-05-08

**Authors:** Jennifer Shuldiner, Ying Liu, Aisha Lofters

**Affiliations:** 1 University of Toronto, 155 College Street, Toronto, ON M5T 1P8 Canada; 2 Institute for Clinical Evaluative Sciences, 2075 Bayview Ave, Toronto, ON M4N 3M5 Canada; 3 Li Ka Shing Knowledge Institute, 209 Victoria St, Toronto, ON M5B 1T8 Canada

**Keywords:** Immigrant, Cancer incidence, Standardized incidence ratio, Breast cancer, Colorectal cancer

## Abstract

**Background:**

Studies have shown that morbidity and mortality rates due to cancer among recent immigrants are lower than those among the native-born population. The objectives of this study were to describe the incidence of colorectal and breast cancer among immigrants from major regions of the world compared to Canadian-born residents of the province of Ontario and to examine the role of length of stay and neighborhood income.

**Methods:**

Retrospective cohort study including all individuals 18 years and over residing in Ontario from 2004 to 2014. Age-standardized incidence rates (ASIR) were calculated for immigrants from each world region versus Canadian-born residents and stratified by neighborhood income quintile and length of stay. Binomial regression analysis was used to determine the association of neighbourhood income, length of stay, and location of birth with colorectal and breast cancer incidence.

**Results:**

Canadian immigrants born in South Asia had the lowest colorectal and breast cancer incidence (colorectal: women: ASIR = 0.14; men: ASIR = 0.18; breast: ASIR = 1.00) compared to long-term residents during the study period (colorectal: women: ASIR = .57; men: ASIR = .72; breast cancer ASIR = 1.61). In multivariate analyses, neighboorhood income did not consistently play a significant role in colorectal cancer incidence; however higher neighbourhood income was a risk factor for breast cancer among immigrant women (adjusted relative risk for highest neighboorhood income quintile versus lowest income quintile =1.21, 95% CI = 1.18–1.24). Increased length of stay was associated with higher risk of cancer. After adjusting for age, neighborhood income, and length of stay, those born in Europe and Central Asia had the highest risk of colorectal cancer compared to those born in East Asia and Pacific and those born in the Middle East had the greatest additional risk of breast cancer.

**Conclusions:**

After correcting for age, breast and colorectal cancer incidence rates among immigrants differ according to their region of birth and recent immigrants to Ontario have lower colorectal and breast cancer incidence rates than their native-born peers. However, those advantages diminish over time. These findings call for Ontario to generate tools and interventions to maintain the health of the immigrant population, particularly for those groups with a higher incidence of cancer.

## Background

Immigrants represent a large, increasing and vital segment of the Canadian population. Most Canadian studies have shown that morbidity and mortality rates of chronic disease among recent immigrants are lower than those among the general Canadian population suggesting that immigrants enjoy the “healthy immigrant effect” whereby they are in better physical condition on arrival than host country inhabitants as a result of selective migration [1–4].

Ontario, the largest province in Canada, has a large and diverse immigrant population and approximately one-third of the population in Ontario is foreign-born [5]. Ontario also has a provincial cancer registry that includes data on all residents diagnosed with cancer and universal public health care coverage, thus making it an ideal location to explore cancer incidence among immigrants at the population level. However, there has been little recent research examining cancer incidence in the immigrant population [6].

Colorectal cancer is the third most common cancer diagnosed in men and women with 26,800 cases per year in Ontario. Breast cancer is the most common cancer among women with 26,300 cases a year in Ontario [7]. Also, colorectal and breast cancer have clear provincial screening guidelines and are often used as indicators for population health [8]. Therefore, the overall objective of this study was to examine how the incidence of colorectal and breast cancer among immigrants from major regions of the world compare to Canadian-born residents of Ontario. We also explored the roles of age, gender, socioeconomic status and time in Canada.

## Methods

The following datasets were linked using unique encoded identifiers and analyzed at the Institute for Clinical Evaluative Sciences (ICES). The Registered Persons Database was used to identify people aged 18 years and over in the province of Ontario eligible for health care. The Registered Persons Database contains basic demographic information for those who have ever received an Ontario health card number for the province’s universal health care system (overall linkage rate = 96.5%). All citizens and permanent residents are eligible for health care. The second database was the Immigration Refugee and Citizenship Canada (IRCC) Database [9] which includes individuals who have landed immigrant or permanent resident status at any time from 1985 to 2014. Immigrants were defined as those identified in the IRCC Database, and long-term residents were defined as those not on the IRCC database (Canadian-born and immigrants who arrived before 1985). The IRCC database was also used to identify country of birth, and countries were further collapsed into eight regions, broadly defined according to the World Bank classification (1, Caribbean and Latin America; 2, East Asia and Pacific; 3, Eastern Europe and Central Asia; 4, Middle East and North Africa; 5, South Asia; 6, Sub- Saharan Africa; 7, USA, Australia, and New Zealand; and 8, Western Europe). Third, we identified incident breast and colorectal cancer cases by linking the cohort to the Ontario Cancer Registry from 2004 to 2014. The Ontario Cancer Registry is a passive surveillance patient registry that links data from hospitals, cancer centers and pathology laboratories; incidence data has been previously assessed as having approximately 92% completeness [10]. The Canadian Census was used for calculating the Canadian population standard.

### Covariates

Using the postal-code conversion file [11], ecological-level measures of income status were estimated using data from the 1996, 2001 and 2006 Canadian census and applied to individual cases according to the dissemination area where the individual resided. Dissemination areas are the smallest geographic census unit for which census data are available, and are uniform in population size, which is targeted from 400 to 700 persons. Individuals were then grouped into income quintiles ranging from 1 (20% lowest income) to 5 (20% highest income). Length of stay was measured by calculating the time since immigration until December 31, 2014 or cancer incidence.

### Analysis

The age-standardized annual incidence rates (ASIR) were calculated using the 2010 Canadian population as standard, for long-term residents, for immigrants, and then by world region of origin for immigrants. To assess the effect of neighboorhood income and length of stay in Canada, ASIR were stratified by time since immigration 0–5 years, 6–10 years and 11+ years) and by neighborhood income quintile (1 through 5).

Predictors of breast and colorectal cancer incidence among all residents in Ontario, 2004–2014 were assessed by two binomial regression models, one among the entire cohort and one among only immigrants. Among the entire cohort, predictors entered into the model included age, place of birth and neighborhood income quintile. The second model calculated among only immigrants assessed the effect of age, neighborhood income quintile, length of stay and region of birth. The analyses produced adjusted rate ratios (RR) with corresponding confidence intervals (CI). Statistical significance was determined at the 0.05 level. All analyses were conducted using SAS statistical software, version 9.4. This study was approved by the institutional review board at Sunnybrook Health Sciences Centre, Toronto, Canada.

## Results

Demographic characteristics of the study population are shown in Table 1. Immigrants were younger than long-term residents on average: mean age ranged from 40.2 ± 13.7 for Sub-Saharan Africa to 44.7 ± 15.8 for East Asia and Pacific, whereas long-term residents’ mean age was 47.5 ± 2. Those that were born in the Middle East and North Africa had spent the least amount of time in Canada on average (10.7 ± 6.0 years), and those born in Europe and Central Asia had spent the longest amount of time (14.2 ± 6.9). Sub-Saharan Africa, followed by Latin America and the Caribbean, had the greatest percentage of immigrants living in the lowest income quintile (Table 1).

**Table 1:** Demographic characteristics of long-term residents and immigrants in the study population

Characteristic	Long-term residents	East Asia and Pacific	Europe and Central Asia	Latin America and the Caribbean	Middle East and North Africa	South Asia	Sub-Saharan Africa	US, New Zealand and Australia
	*N* = 94,136,709	*N* = 5,235,458	*N* = 3,789,083	*N* = 2,718,788	*N* = 1,844,075	*N* = 4,613,474	*N* = 1,141,050	*N* = 380,564
Sex (%)								
Male	48.8	44.9	49.3	47.8	53.1	50.5	49.9	52.0
Age (years)								
Mean (SD)	47.5 ± 3	44.7 ± 15.8	43.1 ± 15.0	42.1 ± 14.8	40.8 ± 14.8	42.0 ± 15.1	40.2 ± 13.7	42.3 ± 15.1
Neighborhood income quintile (%)								
1 (lowest)	17.9	25.1	22.4	33.5	25.7	31.9	43.2	15.3
2	19.5	24.9	19.4	24.7	18.7	24.8	19.6	16.8
3	19.8	19.9	19.5	19.9	19.5	21.5	14.9	18.0
4	21.1	17.5	21.6	13.7	20.5	14.6	12.8	20.2
5 (highest)	21.8	12.4	16.9	7.92	15.2	7.0	9.2	29.4
Length of stay (years) (%) Mean (SD)		12.2 ± 6.4	13.9 ± 6.6	14.2 ± 6.9	11.5 ± 6.4	10.7 ± 6.0	12.4 ± 6.6	12.6 ± 7.0
0–5		16.3	11.4	12.9	19.4	20.4	17.0	18.3
6–10		60.3	68.9	67.0	55.2	49.9	60.3	59.6
11+		23.4	19.7	17.2	25.4	29.7	22.7	22.1

### Place of birth

**Figure 1:**
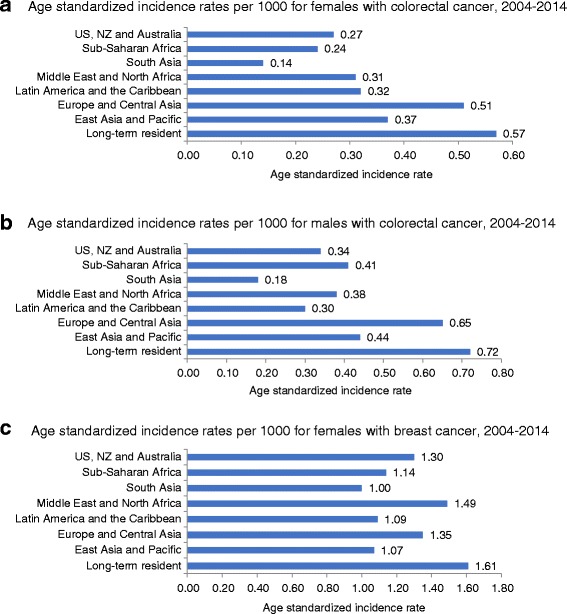
**a**: Age standardized incidence rates per 1000 for females with colorectal cancer, 2004–2014. **b**: Age standardized incidence rates per 1000 for males with colorectal cancer, 2004–2014. **c**: Age standardized incidence rates per 1000 for females with breast cancer, 2004–2014

Age-standardized incidence rates varied by region, with long-term residents consistently having the highest rates and South Asian immigrants consistently having the lowest rates of colorectal and breast cancer (Fig. 1). Among immigrants, incidence of colorectal cancer was highest among Europe and Central Asia for men (ASIR = 0.65) and females (ASIR = 0.51) (Fig. 1). Among women born outside of Canada, the highest ASIR for breast cancer was among those from Middle East and North Africa (ASIR = 1.49, Fig. 1).

### Length of stay and income

**Figure 2:**
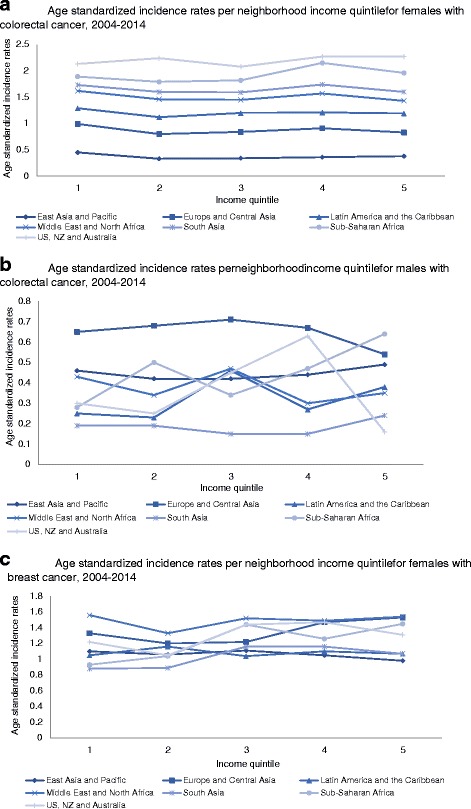
**a**: Age- standardized incidence rates per neighborhood income quintile for female colorectal cancer, 2004–2014. **b**: Age- standardized incidence rates per neighborhood income quintile for male colorectal cancer, 2004–2014. **c**: Age- standardized incidence rates per neighborhood income quintile for female breast cancer, 2004–2014

ASIR were also examined based on length of stay and neighborhood income quintile. We found that the ASIR were not associated with neighborhood income quintile for females and males with colorectal cancer (Fig. 2a and Fig. 2b). Standardized incidence rates of breast cancer increased for higher neighborhood income quintiles for those born in Europe and Central Asia, South Asia, Sub-Saharan Africa, and New Zealand, Australia and the United States, but did not show trends for the remaining regions (Fig. 2c). There were no clear patterns seen for length of stay for both colorectal and breast cancer in the descriptive analysis and advantages enjoyed by immigrants appeared to disappear after spending over 10 years in Canada for both colorectal and breast cancer incidence (not shown).

In the binomial regression analysis among both long-term residents and immigrants we found that, after controlling for age and neighborhood income, immigrants enjoyed a healthy immigrant effect and were at lower risk of breast and colorectal cancer compared to long-term residents (Table 2). For colorectal cancer, those in the highest neighborhood income quintile had a lower risk of incident cancer compared to those in the lowest neighborhood income quintile for both men (RR = 0.96, 95% CI = 0.93–0.99) and women (RR = 0.95, 95% CI = 0.92–0.99). Also, a significant (*p* < 0.01) trend was found for income for colorectal cancer where risk was higher among those in lower income neighborhoods. The effect of neighborhood income on the risk of breast cancer for women was in the opposite direction with each neighborhood income quintile conferring additional risk of breast cancer (RR = 1.21 for Q5 versus Q1, CI = 1.18–1.24), and this relationship was significant as a trend as well (*p* < 0.0001). After adjusting for age and neighborhood income, those born in Europe and Central Asia had the greatest additional risk of colorectal cancer compared to long-term residents. Regarding breast cancer, those born in the Middle East and North Africa had the greatest additional risk of colorectal cancer compared to long-term residents. Those born in South Asia had the lowest rates of breast and colorectal cancer compared to long-term residents (Table 2).

**Table 2:** Multivariate model for entire cohort by cancer site. Variables included in the model are age, sex (for colorectal cancer), income and region of birth

Cancer	Sex	Covariate	RR (95% CI)^a^	*p*-value
**Colorectal Cancer**	**Male**	**Age** ^**b**^	1.41 (1.40, 1.41)	**<.0001**
		**Income**		
		1 (lowest)	1.00	
		2	1.04 (1.01, 1.08)	0.0062*
		3	1.03 (1.00, 1.06)	0.08
		4	1.02 (0.99, 1.05)	0.22
		5 (highest)	0.96 (0.93, 0.99)	**0.0107**
		**Place of birth**		
		Long term resident	1.00	
		East Asia and Pacific	0.57 (0.53, 0.61)	**<.0001**
		Europe and Central Asia	0.87 (0.80, 0.93)	**<.0001**
		Latin America and the Caribbean	0.42 (0.36, 0.47)	**<.0001**
		Middle East and North	0.52 (0.46, 0.59)	**<.0001**
		South Asia	0.26 (0.23, 0.29)	**<.0001**
		Sub-Saharan Africa	0.52 (0.43, 0.63)	**<.0001**
		USANZ	0.47 (0.34, 0.64)	**<.0001**
**Colorectal Cancer**	**Female**	**Age** ^**b**^	1.40 (1.35, 1.36)	**<.0001**
		**Income**		
		1	1.00	
		2	1.01 (0.98, 1.05)	0.40 *
		3	1.00 (0.96, 1.04)	0.97
		4	0.98 (0.95, 1.01)	0.34
		5	0.95 (0.92, 0.99)	0.0059
		**Place of birth**		
		Long term resident	1.00	
		East Asia and Pacific	0.64 (0.60, 0.69)	**<.0001**
		Europe and Central Asia	0.86 (0.79, 0.92)	**<.0001**
		Latin America and the	0.58 (0.52, 0.65)	**<.0001**
		Middle East and North	0.57 (0.48, 0.66)	**<.0001**
		South Asia	0.27(0.23, 0.30)	**<.0001**
		Sub-Saharan Africa	0.44 (0.35, 0.6)	**<.0001**
		USANZ	0.56 (0.41, 0.75)	**0.0001**
**Breast Cancer**	**Female**	**Age** ^**b**^	1.21 (1.21, 1.21)	**<.0001**
		**Income**		
		1	1.00	
		2	1.07 (1.04, 1.09)	**<.0001** *
		3	1.12 (1.09, 1.14)	**<.0001**
		4	1.17 (1.14, 1.19)	**<.0001**
		5	1.21 (1.18, 1.24)	**<.0001**
		**Place of birth**		
		Long term resident	1.00	
		East Asia and Pacific	0.74 (0.71, 0.77)	**<.0001**
		Europe and Central Asia	0.90 (0.86, 0.94)	**<.0001**
		Latin America and the	0.74 (0.70, 0.79)	**<.0001**
		Middle East and North	0.96 (0.90, 1.03)	0.25
		South Asia	0.64 (0.60, 0.67)	**<.0001**
		Sub-Saharan Africa	0.73 (0.66, 0.80)	**<.0001**
		USANZ	0.86 (0.75, 0.97)	0.018

^a^Binomial regression model

^b^per 5 years

^*^P value of trend *p* < 0.005

Bold values are <0.05

In binomial regression analysis among immigrants only, when controlling for age, neighborhood income, and place of birth, we found that the risk of colorectal and breast cancer increased for each additional five years that immigrants lived in Canada (Table 3). Length of stay had the greatest effect on risk of breast cancer where risk increased 7% for each additional five years in Canada (*p* < 0.0001). After adjusting for age, neighborhood income, and length of stay, those born in Europe and Central Asia had the greatest additional risk of colorectal cancer compared to the reference group for this analysis of those born in East Asia and the Pacific. Those born in South Asia had the lowest risk for colorectal cancer among males and females and of breast cancer compared to those born in East Asia and the Pacific (Table 3).

**Table 3:** Multivariate model for immigrant cohort by cancer site. Variables included in the model are age, sex (for colorectal cancer), income, region of birth and length of stay

**Cancer**	**Sex**	**Covariate**	**RR (95% CI)** ^**b**^	***p*** **-value**
**Colorectal Cancer**	**Male**	**Age** ^**a**^	1.38 (1.36, 1.39)	**<.0001**
		**Income**		
		1	1.00	
		2	0.98 (0.88, 1.10)	0.76
		3	1.06 (0.95, 1.19)	0.29
		4	1.01 (0.90, 1.14)	0.86
		5	1.00 (0.88, 1.14)	0.98
		**Place of birth**		
		East Asia and Pacific	1.00	
		Europe and Central Asia	1.45 (1.32, 1.60)	**<.0001**
		Latin America and the Caribbean	0.70 (0.61, 0.80)	**<.0001**
		Middle East and North Africa	0.90 (0.78, 1.03)	0.12
		South Asia	0.45 (0.40, 0.51)	**<.0001**
		Sub-Saharan Africa	0.88 (0.72, 1.06)	0.17
		US, Australia and New Zealand	0.80 (0.58, 1.09)	0.16
		**Length of stay** ^**a**^	1.05 (1.02, 1.08)	**0.0025**
**Colorectal Cancer**	**Female**	**Age** ^**a**^	1.35 (1.33, 1.36)	**<.0001**
		**Income**		
		1	1.00	
		2	0.92 (0.82, 1.03)	0.15
		3	0.93 (0.82, 1.04)	0.21
		4	1.02 (0.90, 1.15)	0.81
		5	0.93 (0.81, 1.06)	0.27
		**Place of birth**		
		East Asia and Pacific	1.00	
		Europe and Central Asia	1.31 (1.19, 1.45)	**<.0001**
		Latin America and the Caribbean	0.89 (0.78, 1.01)	0.062
		Middle East and North Africa	0.88 (0.74, 1.04)	0.12
		South Asia	0.42 (0.36, 0.49)	**<.0001**
		Sub-Saharan Africa	0.67 (0.54, 0.85)	**0.0008**
		US, Australia and New Zealand	0.86 (0.63, 1.16)	0.31
		**Length of stay** ^**a**^	1.05 (1.01, 1.08)	**0.0046**
**Breast Cancer**	**Female**	**Age** ^**a**^	1.19 (1.18, 1.19)	**<.0001**
		**Income**		
		1 (lowest)	1.00	
		2	1.00 (0.94, 1.06)	0.99 *
		3	1.10 (1.03, 1.16)	**0.0021**
		4	1.13 (1.06, 1.19)	**0.0001**
		5 (highest)	1.13 (1.06, 1.20)	**0.0004**
		**Place of birth**		
		East Asia and Pacific	1.00	
		Europe and Central Asia	1.19 (1.12, 1.25)	**<.000**1
		Latin America and the Caribbean	0.96 (0.90, 1.03)	0.22
		Middle East and North Africa	1.29 (1.20, 1.39)	**<.0001**
		South Asia	0.86 (0.81, 0.91)	**<.0001**
		Sub-Saharan Africa	0.95 (0.86, 1.05)	0.35
		US, Australia and New Zealand	1.14 (0.99, 1.30)	0.06
		**Length of stay** ^**a**^	1.07 (1.05, 1.08)	**<.0001**

^a^per 5 years

^b^binomial regression models

*P value of trend p < 0.005

Bold values are <0.05

## Discussion

Our results demonstrate several important findings regarding immigrant health and cancer incidence in Ontario. First, our multivariate regression analyses showed that the healthy immigrant effect exists for recent immigrant arrivals for breast and colorectal cancer incidence but that it dissipated with time and each year in Canada is associated with a 5–7% increase in risk. Second, our study demonstrated that place of birth was an important predictor, with those from Europe and Central Asia being at highest risk among immigrants of developing colorectal cancer (incidence among men = .65 and females =0.51) and those from South Asia having the lowest rates (females = 0.14 and males = 0.18). Those from the Middle East and North Africa were at highest risk among immigrant women to develop breast cancer (incidence = 1.49) and those from South Asia were the lowest (incidence- = 1.00). Third, we saw that neighborhood income did not play a role in colorectal cancer incidence but that higher neighborhood income was a risk factor for breast cancer incidence among immigrant women (RR = 1.21 95% CI = 1.18, 1.24).

Upon examining place of birth, we saw large differences (49–264% difference) in age standardized rates between places of birth. In comparison to long-term residents the largest differences were for those born in South Asia who had the lowest rates for colorectal and breast cancer. These differences are most likely attributed to differences in their home countries where South Asia has among the lowest incidence rates compared to other regions for breast and colorectal cancer [12]. Age-standardized incidence rates for United States, New Zealand and Australia were lower for breast and colorectal cancer compared to long-term immigrants. This was surprising, as Canada is considered to be a historically high-risk area for colorectal and breast cancer, similar New Zealand, United States and Canada, reflecting similar dietary and lifestyle factors [13].

Neighborhood income did not play a significant role in colorectal cancer incidence. Those in the highest neighborhood income quintile had slightly lower rates of colorectal cancer in the regression model including all Ontario residents; however, no effect was seen when examining rates among only immigrants. In contrast, we saw that higher neighborhood income was a risk factor for breast cancer incidence, both unadjusted and in regression models. Similarly, Canadian and American data have shown that women in neighborhoods with higher neighborhood incomes have a higher risk of developing breast cancer [14, 15].

An important dimension of the healthy immigrant effect is that the immigrant advantage we found appeared to disappear after spending over 10 years in Canada for both colorectal and breast cancer incidence. In addition, in the regression model limited to immigrants we saw that risk of cancer increases (5–7%) for each additional five years in Canada for both colorectal and breast cancer. Similarly, researchers have previously found that among Ontarian immigrants, despite the original advantage with immigration, there is a steady decline in survival, and cancer-specific survival, over time [16]. Analysis of Statistics Canada’s Longitudinal Survey of Immigrants to Canada showed a decline in self-assessed health, physical health, and mental health among immigrants as little as two years after arrival [17]. Some researchers believe that convergence in health outcomes may stem from the process of acculturation where immigrants begin to take on Canadian habits such as smoking, alcohol consumption and diet [18]. However, Canadian longitudinal data from representative surveys have shown that immigrants did not show higher rates of daily smoking initiation, however, they were much more likely than the Canadian-born population to have had a substantial weight gain since immigrating [19]. Though these habits may be influential, it is unlikely that they are responsible for changes in incidence occurring over the short time observed in this study.

Others maintain that worsening of health status is due to barriers to health services including lack of familiarity with the Canadian health system and language or cultural differences [20] that may lead to the underuse of preventative health screening and treatment of health problems. To that effect, disparities in cervical and breast cancer screening for foreign-born women have long been documented in Ontario and Canada [21–26]. Similarly, research has shown that cancer incidence may increase in the first decade after immigration and it reaches the population level in the host country in 1–2 generations [27, 28].

This large cohort study has examined cancer incidence among immigrants in Canada which has not been examined since the 1990s [6]. Due to accurate databases, universal health care and excellent linkage we could consider the effects of region of birth, neighborhood income and length of stay. However, there are several limitations that should be noted. We were not able to determine if this was a cohort effect where those that immigrated more recently were healthier than those who immigrated over ten years ago. Our analyses used the world-region-of-birth; this method of grouping could be problematic as countries within any region are not homogeneous. Given the absence of individual-level income-related information, we linked residential postal codes to neighborhood -level income which may have misclassification errors in geocoding [11] in rural areas, however, according to our data 99% of all immigrants to Canada settle in urban areas. Additionally, immigrants may have lived in other countries other than their country of birth before they came to Canada, thus possibly reducing the significance of birthplace as a determinant. Finally, our analysis was not able to account for risk factors such as behaviour (i.e. smoking and alcohol) and stress for cancer.

## Conclusions

Our analysis showed breast and colorectal cancer incidence rates among immigrants to Ontario, Canada are lower than residents and these rates differ according to region of birth, however, those advantages diminish after arrival. Results from this hypothesis-generating research initiative hold significant immigration and health policy implications, and add further intricacy to the study of the social determinants of health. The results call for Ontario to generate tools and interventions to maintain the health of immigrant population.

## Abbreviations

ASIR: age-standardized incidence rates
CI: Confidence Intervals
RR: Rate ratios

## References

[1] Chen J, Ng E, Wilkins R (1996). The health of Canada's immigrants in 1994-95. Health Re.

[2] Cormier RA, Dell CA, Poole N (2003). Women’s health surveillance report. A multi-dimensional look at the health of Canadian women.

[3] Singh GK, Miller BA (2004). Health, life expectancy, and mortality patterns among immigrant populations in the United States. Can J Public Health.

[4] De Maio FG (2010). Immigration as pathogenic: a systematic review of the health of immigrants to Canada. Int J Equity Health.

[5] Statistics Canada. 2011 National Household Survey. Statistics Canada Catalogue no. 99-004-XWE. Ottawa. 2013. http://www12.statcan.gc.ca/nhs-enm/2011/dp-pd/prof/index.cfm?Lang=E. (Accessed 3 May 2018).

[6] McDermott S, DesMeules M, Lewis R, Gold J, Payne J, Lafrance B, Vissandjée B, Kliewer E, Mao Y (2011). Cancer incidence among Canadian immigrants, 1980–1998: results from a national cohort study. J Immigr Minor Health.

[7] Canadian Cancer Statistics 2017 (2017). Canadian Cancer Society’s advisory committee on Cancer statistics.

[8] Papanicolas I, Smith P. Health system performance comparison: an agenda for policy, information and research: an agenda for policy, information and research. McGraw-Hill Education (UK); 2013.

[9] Chiu M, Lebenbaum M, Lam K, Chong N, Azimaee M, Iron K, Manuel D, Guttmann A (2016). Describing the linkages of the immigration, refugees and citizenship Canada permanent resident data and vital statistics death registry to Ontario’s administrative health database. BMC Med Inform Dec Making.

[10] Robles SC, Marrett LD, Clarke EA, Risch HA (1988). An application of capture-recapture methods to the estimation of completeness of cancer registration. J Clin Epidemiol.

[11] Statistics Canada. Postal CodeOM Conversion File Plus (PCCF+) Version 6B, Reference Guide. November 2014 Postal Codes. Statistics Canada Catalogue no. 82-E0086-XDB. Ottawa: Minister of Industry; 2015.

[12] Global Burden of Disease Cancer C (2017). Global, regional, and national cancer incidence, mortality, years of life lost, years lived with disability, and disability-adjusted life-years for 32 cancer groups, 1990 to 2015: a systematic analysis for the global burden of disease study. JAMA Oncol.

[13] Jemal A, Center MM, DeSantis C, Ward EM (2010). Global patterns of cancer incidence and mortality rates and trends. Cancer Epidemiol Prev Biomark.

[14] Borugian MJ, Spinelli JJ, Abanto Z, Xu CL, Wilkins R (2011). Breast cancer incidence and neighbourhood income. Health Rep.

[15] Robert SA, Trentham-Dietz A, Hampton JM, McElroy JA, Newcomb PA, Remington PL (2004). Socioeconomic risk factors for breast cancer: distinguishing individual-and community-level effects. Epidemiology.

[16] Cheung MC, Earle CC, Fischer HD, Camacho X, Liu N, Saskin R, Shah BR, Austin PC, Singh S. Impact of Immigration Status on Cancer Outcomes in Ontario, Canada. J Oncol Pract. 2017:JOP. 2016;13(7):e602–12. 10.1200/JOP.2016.019497 28605254

[17] Newbold B (2009). The short-term health of Canada's new immigrant arrivals: evidence from LSIC. Ethn Health.

[18] Hyman I (2001). Immigration and health. Health policy working paper 01–05.

[19] Ng E, Wilkins R, Gendron F, Berthelot JM. Dynamics of Immigrants' Health in Canada: Evidence from the National Population Health Survey. Ottawa: Statistics Canada; 2005.

[20] Leclere FB, Jensen L, Biddlecom AE. Health care utilization, family context, and adaptation among immigrants to the United States. J Health Soc Behav. 1994:370–84. 7844331

[21] Lofters AK, Moineddin R, Hwang SW, Glazier RH (2011). Predictors of low cervical cancer screening among immigrant women in Ontario, Canada. BMC Womens Health.

[22] Lofters A, Glazier RH, Agha MM, Creatore MI, Moineddin R (2007). Inadequacy of cervical cancer screening among urban recent immigrants: a population-based study of physician and laboratory claims in Toronto, Canada. Prev Med.

[23] Sun Z, Xiong H, Kearney A, Zhang J, Liu W, Huang G, Wang PP (2010). Breast cancer screening among Asian immigrant women in Canada. Cancer Epidemiol.

[24] Vahabi M, Lofters A, Kumar M, Glazier RH (2015). Breast cancer screening disparities among urban immigrants: a population-based study in Ontario, Canada. BMC Public Health.

[25] Wilkins K, Shields M (2009). Colorectal cancer testing in Canada-2008. Health Rep.

[26] Shields M, Wilkins K (2009). An update on mammography use in Canada. Health Rep.

[27] Thomas DB, Karagas MR (1987). Cancer in first and second generation Americans. Cancer Res.

[28] Ziegler RG, Hoover RN, Pike MC, Hildesheim A, Nomura AM, West DW, Wu-Williams AH, Kolonel LN, Horn-Ross PL, Rosenthal JF (1993). Migration patterns and breast cancer risk in Asian-American women. J Natl Cancer Inst.

